# Homology modeling and epitope prediction of Der f 33

**DOI:** 10.1590/1414-431X20186213

**Published:** 2018-03-15

**Authors:** Feixiang Teng, Jinxia Sun, Lili Yu, Qisong Li, Yubao Cui

**Affiliations:** 1 Department of Basic Medicine, Jiangsu Vocational College of Medicine, Yancheng, China; 2 Department of Clinical Laboratory, Wuxi People's Hospital Affiliated to Nanjing Medical University, Wuxi, China

**Keywords:** Der f 33, Homology modeling, B-cell epitope, T-cell epitope, Prediction

## Abstract

*Dermatophagoides farinae* (Der f), one of the main species of house dust mites, produces more than 30 allergens. A recently identified allergen belonging to the alpha-tubulin protein family, Der f 33, has not been characterized in detail. In this study, we used bioinformatics tools to construct the secondary and tertiary structures and predict the B and T cell epitopes of Der f 33. First, protein attribution, protein patterns, and physicochemical properties were predicted. Then, a reasonable tertiary structure was constructed by homology modeling. In addition, six B cell epitopes (amino acid positions 34–45, 63–67, 103–108, 224–230, 308–316, and 365–377) and four T cell epitopes (positions 178–186, 241–249, 335–343, and 402–410) were predicted. These results established a theoretical basis for further studies and eventual epitope-based vaccine design against Der f 33.

## Introduction

House dust mites (HDM), particularly *Dermatophagoides farinae* (Der f) and *Dermatophagoides pteronyssinus* (Der p), are responsible for sensitization of more than 50% of allergic patients worldwide (1,2). Allergens from HDM (fecal material, secretions, body degradation products, and lysates of carcasses) can cause bronchial asthma, atopic dermatitis, and rhinitis (3).

Allergen specific immunotherapy (SIT) is one of the most effective treatments for allergic diseases (4). SIT can be improved by using recombinant allergens, which contain most of the IgE-binding epitopes of the source allergens and are pure and better standardized compared to natural allergen extracts (5). A number of recombinant dust mite allergens have been cloned, expressed, and purified, including Der f groups 1–3, 5–8, 10, 11, 13–18, 22, 24, and 33 allergens (6,7). Allergen extracts of HDM have been used for diagnosis and treatment of IgE-mediated allergic diseases. However, these crude extracts include some inflammatory molecules, such as kallikreins, ceramides, and endotoxins, which could modify treatment outcomes and efficacy (8). Thus, these extracts have some limitations in both their safety and efficacy in SIT (5).

Some SIT approaches have shifted toward epitope-based vaccine design (9,10). In this approach, a recombinant allergen contains multiple B and T cell epitopes. Thus, identifying the major B and T cell epitopes of allergens is critical for effective immunotherapy of allergic diseases via epitope-based vaccine preparation.

To date, 36 groups of mite allergens have been listed in the Allergen Nomenclature Database (www.allergen.org). Der f 33 was identified in 2014 (GenBank accession KM010005), and it was characterized as having a molecular weight of 52 kDa and belonging to the alpha-tubulin protein family. Moreover, Der f 33 could react to the serum of patients with mite allergy; the positive rate of skin prick test to Der f 33 was 23.5% (4/17 patients). Also, it can modulate the functions of dendritic cells (DCs) and induce airway allergy (7). However, the major B and T cell antigen epitopes of Der f 33 have not been reported.

In this study, we used bioinformatics to predict the secondary and tertiary protein structures and identify the B and T cell epitopes of Der f 33. These findings provide theoretical support for mite allergen epitope-based vaccine design.

## Material and Methods

### Sequence retrieval and analyses

Der f 33 amino acid sequence (Accession Number: AIO08861.1) was obtained from the International Union of Immunological Societies (IUIS) nomenclature database and the protein database of National Center for Biotechnology Information (NCBI). Family classification of Der f 33 was analyzed by Superfamily v1.75 (11) and InterPro v56.0 (12). TMHMM server 2.0 (13) was used for predicting the transmembrane helices in Der f 33 proteins.

### Physicochemical analysis and secondary structure prediction

Physicochemical analysis including molecular weight, negatively charged residues, positively charged residues, theoretical pI, aliphatic index, grand average of hydropathicity (GRAVY), and instability index of Der f 33 was predicted by ProtParam (14). Characteristic patterns and functional motifs of Der f 33 were checked by using Prosite (15). Secondary structure of Der f 33 was predicted by Jpred 4.0 (16).

### Tertiary structure prediction and evaluation

Homology modeling was used for constructing the tertiary structure of Der f 33. BLASTP search was performed against the Protein Data Bank (PDB) to find suitable Der f 33 templates, which were based on the high score, lower e-value, and maximum sequence identity. Tertiary structure was constructed by MODELLER v9.16 (17), which was imported to Chiron (18) to rectify unfavorable clashes and improve the quality of stereochemistry.

Estimating the quality of tertiary structure is a vital step. VERIFY_3D (19) was used to determine the compatibility of an atomic model (3D) with its own amino acid sequence (1D) and compare the results to good structures. PROCHECK (20) was used to check the stereochemical quality of Der f 33 structure. ERRAT (21) was used to analyze the statistics of non-bonded interactions between different atom types. ProSA (22) was used to analyze the Z-score, which shows the degree of match between the template protein and Der f 33. QMEAN (23) is a composite scoring function, which was used to derive both global (for the entire structure) and local (per residue) error estimates based on one single model. Visualization of tertiary structure was performed using UCSF Chimera 1.10.2 (24).

### Prediction of B cell epitopes

ABCpred (25), BCPreds (26), BcePred (27), and Bioinformatics Predicted Antigenic Peptides (BPAP) system (28) were used for predicting B cell epitopes of Der f 33. ABCpred predicted B cell epitopes in antigen sequences, using an artificial neural network. BCPreds selected AAP method (26), BCPred (29), and FBCPred (30) to predict B cell epitopes. BcePred and BPAP system predicted B cell epitopes using the same physicochemical properties, such as hydrophilicity, flexibility/mobility, accessibility, polarity, exposed surface, and turns.

### Prediction of T cell epitopes

T cell epitopes were predicted by identifying the binding of peptides to MHC molecules with NetMHCII 2.2 (31) and NetMHCIIpan-3.1 (32).

NetMHCII 2.2 uses artificial neuron networks to predict binding of epitope peptides to HLA-DQ alleles in regions of HLA-DQA10101-DQB10501, HLA-DQA10102-DQB10602, HLA-DQA10301-DQB10302, HLA-DQA10401-DQB10402, HLA-DQA10501-DQB10201, and HLA-DQA10501-DQB10301.

NetMHCIIpan-3.1 was used for HLA-DR-based epitope prediction in regions of HLA-DR DRB101, HLA-DRB301, HLA-DRB401, and HLA-DRB501.

In the 2 programs, high binding peptides have an IC50 value below 50 nM. The ultimate T cell epitopes were obtained by combining the results of the HLA-DR alleles epitopes and HLA-DQ alleles epitopes.

## Results

### Amino acid sequence analysis

The ProtParam results showed that the complete amino acid sequence of Der f 33 comprises 461 amino acids and has a molecular weight of 51.6 kDa. The number of negatively charged residues (Asp+Glu) and positively charged residues (Arg + Lys) were 62 and 42, respectively. The theoretical pI and aliphatic index of Der f 33 were 5.04 and 79.11, respectively. The GRAVY and instability index were -0.286 and 43.23, respectively.

The results of InterPro v56.0 and Superfamily v1.75 showed that Der f 33 belonged to the alpha-tubulin protein family (InterPro No. IPR002452) and tubulin protein superfamily (InterPro No. IPR000217). Prosite analysis of Der p 33 revealed that it contained a TUBULIN pattern (PS00227, 149–155, GGGTGSG). The computed results of TMHMM Server 2.0 showed that Der f 33 has no transmembrane helices, and the protein sequences are all located outside of the membrane.

### Tertiary structure construction and analysis

As the homology modeling template, Cytotoxic Dolastatin 10 Analogues (PDB accession No.: 4X20) have a high sequence identity (82%), lower e-value (0.0) and a high score (761) with Der f 33.

**Figure 1.**
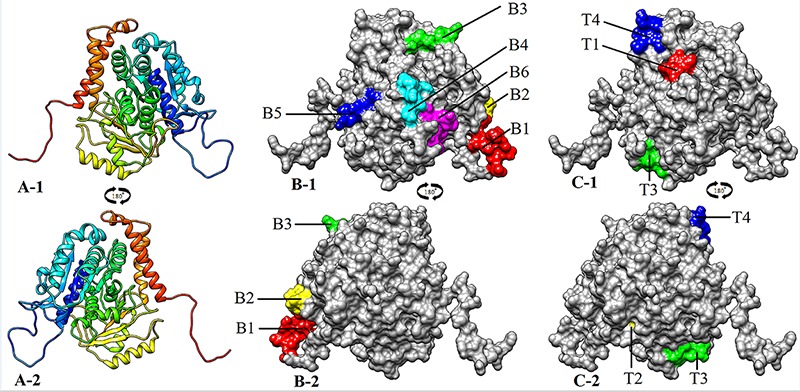
B and T cell epitopes on tertiary structure of Der f 33. *A-1* and *A-2*, Tertiary structure of Der f 33. *B-1* and *B-2*, B cell epitopes on tertiary structure of Der f 33. *C-1* and *C-2*, T cell epitopes on tertiary structure of Der f 33.

The Ramachandran plot of tertiary structure showed that 86.3% amino acid residues of Der f 33 were within the most favored regions, 12.3% of residues were in the additional allowed region, 0.5% residues in the generously allowed regions, and 1.0% residues in the disallowed region. The application of the ERRAT program showed that the overall quality factor is 85.34. VERIFY 3D program revealed that 88.72% of the residues had an averaged 3D-1D score ≥0.2. As indicated by the ProSa server, the Z-scores of Der f 33 and 4X20 are -8.89 and -8.68, respectively. The QMEAN Z-score of Der f 33 was -0.927 and Q value was 0.692 (Table 1). The tertiary structure of Der f 33 is shown in Figure 1.

**Table 1.** Parameters of Der f 33 tertiary structure.

Protein/Structural assessment methods	Ramachandran plot (%)	ERRAT	VERIFY 3D	Z-score	Q value
Der f 33					
PROCHECK analysis	86.3% core	85.337	88.72%		
	12.3% allow				
	0.5% generously				
	1.0% disallowed				
ProSa				−8.89	
QMEAN				−0.927	0.692
4X20					
PROCHECK analysis	83.6% core	83.72	89.28%		
	14.8% allow				
	1.0% generously				
	0.6% disallowed				
ProSa				−8.68	
QMEAN				−1.11	0.652

Core: most favored regions; allow: additional allowed regions; generously: generously allowed regions; disallowed: disallowed regions.

**Figure 2.**
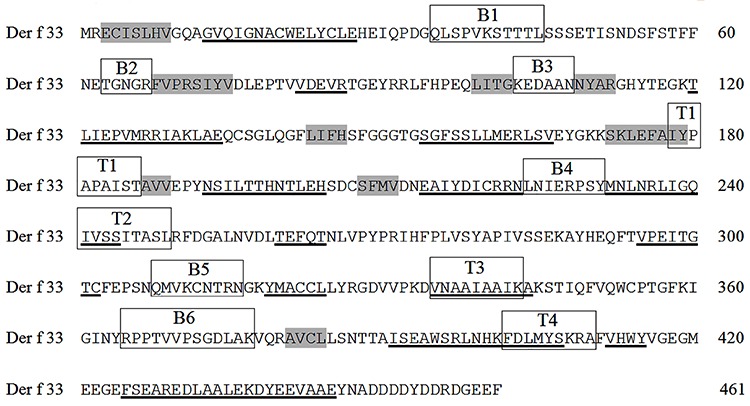
Secondary structure elements for Der f 33. The α-helices are underlined, β-sheets are shown in gray highlight, random coils in unlabeled sequence, and epitopes are within a box.

In the secondary structure of Der f 33, the percentages of overall amino acids located in α-helices, β-sheets, and random coils are 33.41% (14 domains), 9.98% (9 domains), and 56.61%, respectively. The tertiary structure of Der f 33 also contain α-helices, β-sheets, and random coils, and the amino acid numbers of these three elements are slightly different from the secondary structures. The percentages of overall amino acids of tertiary structure located in α-helices, β-sheets, and random coils are 43.17% (17 domains), 14.32% (12 domains), and 42.51%, respectively (Table 2, Figure 2).

**Table 2.** Secondary and tertiary structure elements of Der f 33.

Structure	α-helices (%)	β-sheets (%)	Random coils (%)
Secondary structure	33.41 (14 domains)	9.98 (9 domains)	56.61
Tertiary structure	43.17 (17 domains)	14.32 (12 domains)	42.51

### B cell epitope prediction

Combining the results of four programs, six antigenic epitope peptides (amino acid positions 34–45, 63–67, 103–108, 224–230, 308–316, and 365–377) were predicted (Table 3, Figures 1 and 2).

**Table 3.** Predicted B and T cell epitopes of Der f 33.

Peptide	Type of epitope	Position	Sequence
P1	B	34–45	**G**QL**S**PV**KS**TTTL
P2	B	63–67	T**GNG**R
P3	B	103–108	**K**ED**AAN**
P4	B	224–230	L**N**IERP**S**
P5	B	308–316	QMV**K**C**N**TR**N**
P6	B	365–377	RPPTVVP**SG**DL**AK**
P7	T	178–186	IYP**A**P**A**I**S**T
P8	T	241–249	IV**SS**IT**AS**L
P9	T	335–343	V**NAA**I**AA**IK
P10	T	402–410	FDLMY**SK**R**A**

Bold letters represent the hydrophobic amino acid residues.

### T cell epitope prediction

NetMHCIIpan 3.1 and NetMHCII 2.2 were used for predicting T cell antigenic epitopes. Combining the results of the two programs, the consensus results were for four predicted T cell epitopes (amino acids positions 178–186, 241–249, 335–343, and 402–410) (Table 3, Figures 1 and 2).

## Discussion

HDM are important sources of inhalant and contact allergens that can cause a variety of allergic diseases (3). Thus, molecular characterization and identification of epitopes of HDM allergens will promote a better understanding of immune response and promote an effective epitope-based vaccine design.

To better understand the structure and function of Der f 33, we first analyzed the basic sequence properties. The bioinformatics analyses showed that Der f 33 is a hydrophilic (GRAVY) and unstable (instability index) protein, which has no transmembrane helices, and the protein sequences are all located outside of membrane.

Homology modeling built a target structure based on the comparison with the data extracted from homologous sequences with suitable templates (33). A total 98.6% amino acid residues of Der f 33 were in favored and allowed regions, showing that the distribution of the amino acid is reasonable. The VERIFY 3D and ERRAT results showed that the tertiary structure of Der f 33 was good and had high resolution. The ProSa results showed that there was a high tertiary structure matching degree between Der f 33 protein and the template protein. The standard deviation value of QMEAN Z-score was less than 1, showing that the Der f 33 protein model variation rate was low, the overall folding and local structure both had high accuracy rate, and stereochemistry was reasonable. In addition, the Q value was between 0 and 1, showing that the predicted model of Der f 33 was reliable and could be adopted for this study.

The secondary and tertiary structure of Der f 33 both contain three elements (α-helices, β-sheets, and random coils); the amino acid percentages of these three elements in the tertiary structure differed slightly from the secondary structure. This phenomenon may be due to different methods of prediction for the secondary and tertiary structures.

Hydrophobicity, fragment flexibility/mobility, surface accessibility, polarity, exposed surface, and turns are important features for B cell antigenic epitope identification. These antigenic indexes showed the epitope-forming capacity of the Der f 33 amino acid sequence. Moreover, secondary and tertiary structures are important for B cell epitope prediction. The α-helices and β-sheets have higher chemical bond energy, making epitope formation difficult. Random coils are located in surface-exposed regions of a protein, which often contain epitope sequences (34). Integrating the results from the four programs and combining with the secondary and tertiary structures, the final B cell epitopes included six sequences: amino acid positions 34–45, 63–67, 103–108, 224–230, 308–316, and 365–377. The prediction results showed that T cell epitopes contained four sequences: amino acid positions 178–186, 241–249, 335–343, and 402–410.

Finally, allergen epitopes usually contained high proportion hydrophobic amino acids residues (Ala, Ser, Asn, Gly, and Lys) (35). The prediction results showed that the B and T cell epitopes of Der f 33 both contain multiple hydrophobic amino acids. However, these predicted epitopes require experimental verification.
